# Personalized Medicine in Thoracic Surgery: The Role and Future of Robotic-Assisted Techniques

**DOI:** 10.3390/jpm13060986

**Published:** 2023-06-13

**Authors:** Takashi Eguchi

**Affiliations:** Division of General Thoracic Surgery, Department of Surgery, Shinshu University School of Medicine, Matsumoto 390-8621, Japan; eguchi_t@shinshu-u.ac.jp; Tel.: +81-263-37-3576

The advent of robotic-assisted thoracic surgery (RATS) has revolutionized the field of thoracic surgery, offering a new paradigm for personalized, precision, and individualized medicine. The unique capabilities of RATS, including enhanced dexterity, precision, and visualization, allow for a more tailored approach to each patient’s specific needs. This personalized approach is particularly relevant in managing thoracic malignancies, where the surgical approach can significantly impact patient outcomes [1]. As we continue to advance in the era of precision medicine, the role of RATS is expected to expand, offering more personalized and optimal treatment options for patients with thoracic malignancies. This paper will discuss the current status and future perspectives of RATS, focusing on its role in personalized medicine.

<Innovative robotic approach for personalized lung resection>

Sakakura et al. report a novel approach to robotic lung resection, namely segmentectomy [2]. The authors present a three-arm robotic open-thoracotomy-view approach and detail the port placements, system setting, and the roles of two assistants and confronting monitors in this approach. They emphasize that cranial-side intrathoracic structures, often hidden in the conventional look-up-view method, were well visualized, and these settings enable the console surgeon and the two assistants to have the same views as they naturally do in their open-thoracotomy procedures. This approach may offer a feasible alternative to traditional methods, providing enhanced precision and visualization.

<Tailoring RATS for diverse mediastinal tumor settings>

Building on the concept of personalized surgical approaches, the second paper, from Okazaki et al., discusses the benefits of RATS for different types of mediastinal tumors, including anterior, superior, middle, and posterior mediastinal tumors [3]. The authors emphasize that the position and port placement should be chosen based on the tumor’s size, location, and aggressiveness. The paper also discusses using RATS to resect dumbbell tumors, which require a posterior approach and can be performed in the prone position. The authors suggest that RATS may further expand its indication for mediastinal tumors as robots evolve, emphasizing the importance of personalized medicine in determining the optimal surgical approach for each patient.

<Enhancing lung segmentectomy outcomes with RATS>

The third paper emphasizes the importance of personalized surgery in the era of precision medicine. Eguchi et al. discuss the use of RATS in lung segmentectomy, highlighting the role of RATS in improving operative performance due to its core technological features [4]. The authors discuss the challenges of lung segmentectomy, particularly the need for preoperative planning and intraoperative navigation based on individual segmental topographic anatomy. They suggest that the RATS approach has an advantage in terms of obtaining better surgical visualization due to the magnified visualization using its binocular scope system and the steady retraction using its assistant arm. The authors emphasize the importance of personalized surgery in precision medicine, tailoring the surgical approach to individual patients based on the oncological features of lung tumors, hilar anatomy, functional background, and surgical tolerance, underscoring the potential of RATS to enhance patient outcomes through personalized surgical strategies.

<Evolution of robotic port placement for personalized surgery>

Sakakura and Eguchi discuss the four phases of port placement variations, from the initial phase of mimicking video-assisted thoracic surgery (VATS) to the fourth phase of maximizing the functional features of the da Vinci Xi robotic system [5]. They highlight the advancements of the da Vinci Xi system, including the introduction of the robotic stapler and the improved latitude of robotic arms based on reduced external arm collision and flexibility of port placements. This evolution underscores the adaptability of RATS and its potential for further personalization based on the patient’s unique needs and the surgeon’s preferences. The authors conclude by emphasizing the importance of thoracic surgeons being thoroughly versed in these approaches to choose the one that best suits the case, further reinforcing the role of personalized medicine in RATS.

<Superior noncancer outcomes with RATS in lung cancer treatment>

The final paper, from Zhang et al., compares RATS and open thoracotomy in the context of non-small cell lung cancer treatment [6]. The authors highlight the feasibility and safety of the RATS approach, which offers three-dimensional visualization, enhanced precision, and ergonomic advantages. They present a meta-analysis that reaffirms the superior perioperative outcomes of robotic resections compared to open thoracotomy, including a lower incidence of atrial arrhythmias and a higher number of lymph node stations harvested. This paper underscores the potential of RATS to enhance patient outcomes through personalized surgical strategies, further reinforcing the role of RATS in the era of precision medicine. The authors suggest that further innovation of the robotic platform and improved accessibility and affordability will help consolidate its role in the surgical management of lung cancer.

## Future Perspectives

The future of RATS in thoracic surgery is promising, with ongoing advancements expected to further enhance the precision and personalization of surgical interventions. As technology continues to evolve, we anticipate further improvements in surgical outcomes, patient recovery, and overall quality of life. The integration of artificial intelligence and machine learning into RATS may also offer new opportunities for personalized medicine, potentially enabling more accurate preoperative planning, intraoperative navigation, and postoperative monitoring. As we continue to move forward in this exciting era of precision medicine, the role of RATS in providing personalized and optimal treatment for thoracic malignancies is expected to become increasingly prominent.

## References

[1] Hristov B., Eguchi T., Bains S., Dycoco J., Tan K.S., Isbell J.M., Park B.J., Jones D.R., Adusumilli P.S. (2019). Adusumilli, Minimally Invasive Lobectomy Is Associated With Lower Noncancer-specific Mortality in Elderly Patients: A Propensity Score Matched Competing Risks Analysis. Ann. Surg..

[2] Sakakura N., Nakada T., Takahashi Y., Suzuki A., Shinohara S., Kuroda H. (2022). Three-Arm Robotic Lung Resection via the Open-Thoracotomy-View Approach Using Vertical Port Placement and Confronting Monitor Setting: Focusing on Segmentectomy. J. Pers. Med..

[3] Okazaki M., Shien K., Suzawa K., Sugimoto S., Toyooka S. (2022). Robotic Mediastinal Tumor Resections: Position and Port Placement. J. Pers. Med..

[4] Eguchi T., Miura K., Hamanaka K., Shimizu K. (2022). Adoption of Robotic Core Technology in Minimally Invasive Lung Segmentectomy: Review. J. Pers. Med..

[5] Sakakura N., Eguchi T. (2023). Port Placement Variations for Robotic Lung Resection: Focusing on Their History, Conventional Look-Up-View and Horizontal Open-Thoracotomy-View Techniques, and More. J. Pers. Med..

[6] Zhang O., Alzul R., Carelli M., Melfi F., Tian D., Cao C. (2022). Complications of Robotic Video-Assisted Thoracoscopic Surgery Compared to Open Thoracotomy for Resectable Non-Small Cell Lung Cancer. J. Pers. Med..

